# Topical anesthetics for needle-related pain in adults and children (TOPIC): a mini-review

**DOI:** 10.3389/fpain.2023.1350578

**Published:** 2024-01-08

**Authors:** Sylvie Le May, Wenjia Wu, Maxime Francoeur, Philippe Dodin, Evelyne Doyon-Trottier, Nicole Hung, Estelle Guingo, An Kateri Vu, Annie Sylfra, Laurence Lessard, Stephany Cara-Slavich, Kathryn DeKoven

**Affiliations:** ^1^ Institut TransMedTech, CHU Sainte-Justine Research Center, Montreal, QC, Canada; ^2^ Faculty of Nursing, University of Montreal, Montreal, QC, Canada; ^3^ Faculty of Dental Medicine, University of Montreal, Montreal, QC, Canada; ^4^ Department of Dental Medicine, CHU Sainte-Justine, Montreal, QC, Canada; ^5^ Medical Librairies, Direction de l’enseignement, CHU Sainte-Justine, Montreal, QC, Canada; ^6^ Emergency Department, CHU Sainte-Justine, Montreal, QC, Canada; ^7^ Faculty of Medicine, University of Montreal, Montreal, QC, Canada; ^8^ Department of Creation and New Medias, University of Quebec in Abitibi-Temiscamingue, Rouyn-Noranda, QC, Canada; ^9^ Department of Anesthesiology, CHU Sainte-Justine, Montreal, QC, Canada

**Keywords:** needle-related procedures, topical anesthetic, pain, topical cream, liposomal lidocaine, tetracaine hydrochloride

## Abstract

**Purpose:**

Healthcare professionals (HCP) perform various needle procedures that can be distressing and painful for children and adults. Even though many strategies have been proven effective in reducing distress and pain, topical anesthetic use before needle procedures is uncommon. However, there are limited studies in the existing literature comparing specifically liposomal lidocaine and tetracaine hydrochloride topical creams.

**Source:**

This systematic review analyzed studies on the use of two anesthetic creams, Liposomal Lidocaine (Maxilene®) and Tetracaine hydrochloride (Ametop™), in children and adults undergoing a needle-related procedure. Databases searched: PubMed, CINAHL, ClinicalTrials. Only randomized controlled trials (RCT) and Controlled Clinical Trials (CCT) studies were included. Cochrane Collaboration's Risk of Bias assessment tool was used. Strictly minimally invasive procedures were included to standardize different skin needle interventions.

**Principal findings:**

Only one study with 60 participants was available to be included in this review. No statistically significant difference was found in the mean pain score among both interventions. The outcomes of self-reported distress during cannulation and on HCP satisfaction were not reported. However, physiological characteristics associated with stress/anxiety and on cannulation success rate were reported and did not show statistical significance.

**Conclusion:**

Little to no evidence regarding the most efficient cream between liposomal lidocaine and tetracaine hydrochloride for pain management during needle-related procedures was found. Further studies, particularly RCT with larger sample sizes and standardized outcome measures, are needed to confirm the relative efficacy of either anesthetic cream.

## 1 Introduction

Healthcare professionals (HCP) perform various painful procedures that can be distressing and painful for both children and adults. Among these, needle-related procedures are often required to diagnose and treat illnesses, either through injections or other minimally invasive procedures such as phlebotomy. Pain and distress may decrease collaboration and increase procedural time and number of attempts (1–4). Acute pain felt during needle procedures may cause long-term conditioned anxiety. Recalling past experiences may cause great discomfort to adults and children and emotional distress to parents. People who have experienced painful procedures as a child may be conditioned to experience procedural anxiety as adults (3, 5). Reduction in pain and distress is beneficial to children, parents, and HCP as it increases current and future collaboration (6, 7).

Many strategies have been proven effective in reducing pain and distress during procedures using prevention, physical, psychological, and pharmacological methods. For needle procedures, the topical anesthetic application is the first pharmacological strategy recommended by the Canadian Pediatric Society (2, 8, 9). Unfortunately, the use of topical anesthetic prior to needle procedures is not common practice. In the past, some have reported that less than 10% of phlebotomy or other skin puncture procedures, performed on children, are provided with any topical anesthetic (10). This continues to be relevant to this day. A recent survey of 128 phlebotomists showed that while methods to reduce distress in children (e.g., comfort techniques/positioning) were used 70% of the time, only 40% used distractions (e.g., tablets) and as low as 20% included a child life specialist (CLS) or other pain-reducing methods such as buzzy devices or local anesthetics (11). This survey also reports that half of the surveyed phlebotomists seldom use available resources due to a lack of training in child development (11). While local anesthetic injections have been used in children and adults in the past, topical formulations don't require any needles. Indeed, they are able to decrease needle-related pain and anxiety without additional pain.

Topical anesthetic creams can be an easy-to-use solution for painful needle procedures. After the application to the skin surface and absorption through the skin, the anesthetic effect begins, with an onset of action of 30–60 min (12). The anesthetic effect varies from one hour to four hours depending on the compound used. Depending on their formulation, some creams may be more efficient in reducing pain than others (12), however, the optimal formulation still needs to be determined.

Liposomal Lidocaine cream (Maxilene®) is used as a topical anesthetic cream and works as a reversible sodium-channel nerve blocking agent which prevents pain receptor signaling, causing a loss of sensation within the targeted skin area (12). Liposomal lidocaine cream has a specific formulation using a liposomal encapsulation to protect the lidocaine molecule and reduce its metabolism rate, thus extending its anesthetic effect (13–15). The onset of action is about 30 min, which is faster to lidocaine-prilocaine cream, requiring one hour of application (15, 16). No systemic reactions and only mild skin reactions, such as erythema and irritation, have been reported, making liposomal lidocaine an interesting option for pain management in both children and adults (15). Moreover, it has been reported that liposomal lidocaine produces less vasoconstriction than lidocaine-prilocaine cream which may also help with venipuncture (15). No results have yet to be published on the effect of topical liposomal lidocaine on electrolyte levels when sampled through peripheral phlebotomy or any other methods.

Tetracaine hydrochloride (Ametop™) is a topical anesthetic which also works as a nerve blocking agent by selectively inhibiting ion influx through sodium-channels of the nerve endings within the dermis, preventing pain receptor signaling, thus causing a loss of sensation (12, 17). Tetracaine hydrochloride cream is an ester-type topical anesthetic with a similarly fast onset of action of about 30 min with greater potency given by its lipophilic nature and thus it penetrates the skin faster than regular lidocaine (18). Tetracaine hydrochloride is considered safe as no systemic reaction has been observed in either adults or children. Adverse effects, such as erythema and pruritis beyond the site of application, are rare in clinical settings (17, 18). No results have been published on the effect of topical tetracaine hydrochloride on electrolyte levels when sampled through peripheral phlebotomy, after applying the product on a vein, as recommended (17). In a small study, the local anesthetic was suspected to alter sodium and potassium levels in capillary blood sampling, however, the cream is not meant to be applicated on finger tips (19). Recommendations have been issued to avoid the use of tetracaine hydrochloride during drug administration, such as immunization, to avoid drug on drug interactions (17).

Both creams have independently been shown to efficiently reduce procedural pain (20–22). Yet, there seems to be no consensus on which topical anesthetic is better suited for children and adults. Multiple studies have been performed in the past using one cream or the other against a placebo, a cold and vibrating device (Buzzy) and other anesthetic creams (8, 20–22). However, to the best of our knowledge our systematic review have compared the effects or efficacy of both creams in either children or adults.

Thus, the purpose of this systematic review was to address the following question: Is liposomal lidocaine (Maxilene®) a better topical anesthetic cream than tetracaine HCl (Ametop™) to reduce pain during skin puncture procedures in children and adults? The secondary objectives of this review were to observe self-reported anxiety, satisfaction levels of HCP, parents as well as patients, the occurrence of side effects and success rate of the needle related procedure of both anesthetic creams.

## 2 Methods

### 2.1 Eligibility criteria

This systematic review targets liposomal lidocaine (Maxilene®) and tetracaine hydrochloride (Ametop™) topical anesthetic creams in both children starting at 6 years-old and adults starting at the age of 18 years-old having a needle procedure. Limitation of age was capped at six years old since only one study found that children younger than 6 years could provide a reliable pain intensity estimate (24). Only randomized controlled trials (RCT) and Controlled Clinical Trials (CCT) studies on either anesthetic cream in children or adults or both, of either sex, were included in this review. All hospital units or healthcare settings were included.

To minimize the difference in stimuli from different skin needle procedures, the review was limited to venipuncture, venous cannulation, intramuscular injection, and subcutaneous injection only and excluded more invasive procedures such as abscess drainage. Data from case studies, abstracts, posters, conferences and reports were considered only if additional information was available.

### 2.2 Types of outcomes

#### 2.2.1 Primary outcome

The primary outcome of this study was the difference in mean pain score, between both creams, during any needle-related procedures. All included studies used a known and validated self- reported pain scale such as Numerical Rating Scales [NRS] (25) and Visual Analogue Scale [VAS] (25), Faces Pain Scale- Revised [FPS-R] (26), or an observational pain scale such as Face Legs Activity Cry Consolability Scale [FLACC] (27), or EVENDOL (EValuation ENfant DOuLeur in French, Evaluation Child Pain) pain scale (28).

#### 2.2.2 Secondary outcomes

Secondary outcomes were as follows:

1. Self-reported anxiety and distress (NRS, VAS, and other validated scales)
2. Satisfaction level of HCP (customized satisfaction surveys)
3. Satisfaction of children and parents
4. Occurrence of adverse events
5. Success rate of the needle-related procedure

### 2.3 Information source

This systematic search was performed according to the Preferred Reporting Items for Systematic Review and Meta-Analysis (PRISMA) guidelines (29). The protocol was registered in the PROSPERO database (Registration ID: CRD42022342742, on December 9th 2022).

The search was performed by an independent trained librarian (PD) from the Centre Hospitalier Universitaire (CHU) Sainte-Justine following specific criteria and limited to a period of 20 years (2002–2022) and only to French and English publications. Databases searched included Cochrane Central Register of Controlled trials (CENTRAL), CINAHL, Embase, MEDLINE (Ovid), PubMed and ProQuest. The references list of all identified articles was searched manually, and additional articles identified this way were included and marked as such within the flow chart. The full search strategy is described in the appendix. Unpublished articles and ongoing trials were identified through trials registers (ClinicalTrials.gov) available online. Authors were contacted if additional information was needed.

### 2.4 Data collection

#### 2.4.1 Study selection

Two reviewers (MF, WW) independently screened the retrieved titles and abstracts using the web application *Covidence* to select studies meeting the predetermined inclusion criteria. Reviewers were not blinded to authors and journals during the selection process. Eligibility for inclusion of the full-text studies was assessed for methodological quality. Authors of studies were contacted if additional information was needed. Studies still missing data or not meeting the quality criteria were excluded and reasons were recorded. Any discrepancies and disagreements in the selection process for screening and eligibility were resolved by consensus. If it was not possible, a third review author (SLM) was consulted until consensus was reached.

#### 2.4.2 Data extraction

Studies included in this systematic review were processed by all reviewers to extract data using a standardized form. Study characteristics data were collected for each study as followed: randomization techniques, interventions, quantity/dosage used, control used, area of application, duration of application and any pharmacological or psychological co-interventions such as the presence of a CLS or any other preparation, comfort position, relaxation technique, or distractions used.

#### 2.4.3 Measures of outcomes

For continuous variables, such as pain or distress, mean scores and standard deviations were collected as well as the scale used for means of comparability. For dichotomous variables, such as occurrence of adverse events from the usage of topical creams and the success of the procedure, the number of events were recorded.

#### 2.4.4 Risk of bias

Selected studies were assessed independently by all reviewers for the risk of bias using the Cochrane Collaboration's Risk of Bias assessment tool. The first part of the tool consists in recording the information reported within the studies according to each of the specific domains. The second part consists in scoring the studies according to the *high*, *low* and *unclear* point system on all six domains of bias from the tool (30).

#### 2.4.5 Reporting bias assessment

Any missing data uncovered during the selection process was resolved by contacting the authors of the study in question to obtain the additional information needed. If data remained missing after contacting authors, these studies have not been included and marked as *Missing data* in the flow chart. Bias was assessed using funnel plot analysis of asymmetry (31) and the Egger's test (32), if required.

### 2.5 Data analysis

A meta-analysis was planned as long as at least two different studies report using comparable scales to evaluate outcomes and all analysis was calculated by an independent biostatistician. To evaluate the effects of each intervention, continuous variables were evaluated using mean difference (MD) and a 95% confidence interval (CI). Dichotomous variables were evaluated using a risk ratio (RR) and a 95% CI.

#### 2.5.1 Subgroups' analysis

If a meta-analysis is possible, subgroup analysis was planned to be performed using a chi-square (*χ*^2^) test. Subgroups are as follows:

1. Age: was stratified according to the Eunice Kennedy Shriver National Institute of Child Health and Human Development (NICHD) Pediatric Terminology cited by Williams et al. (33)
2. Needle procedure: if multiple needle procedures are used, analysis was performed according to the type of procedure (intramuscular injection, subcutaneous injection, venipuncture, and venous cannulation).

## 3 Results

### 3.1 Study characteristics

**Figure 1:**
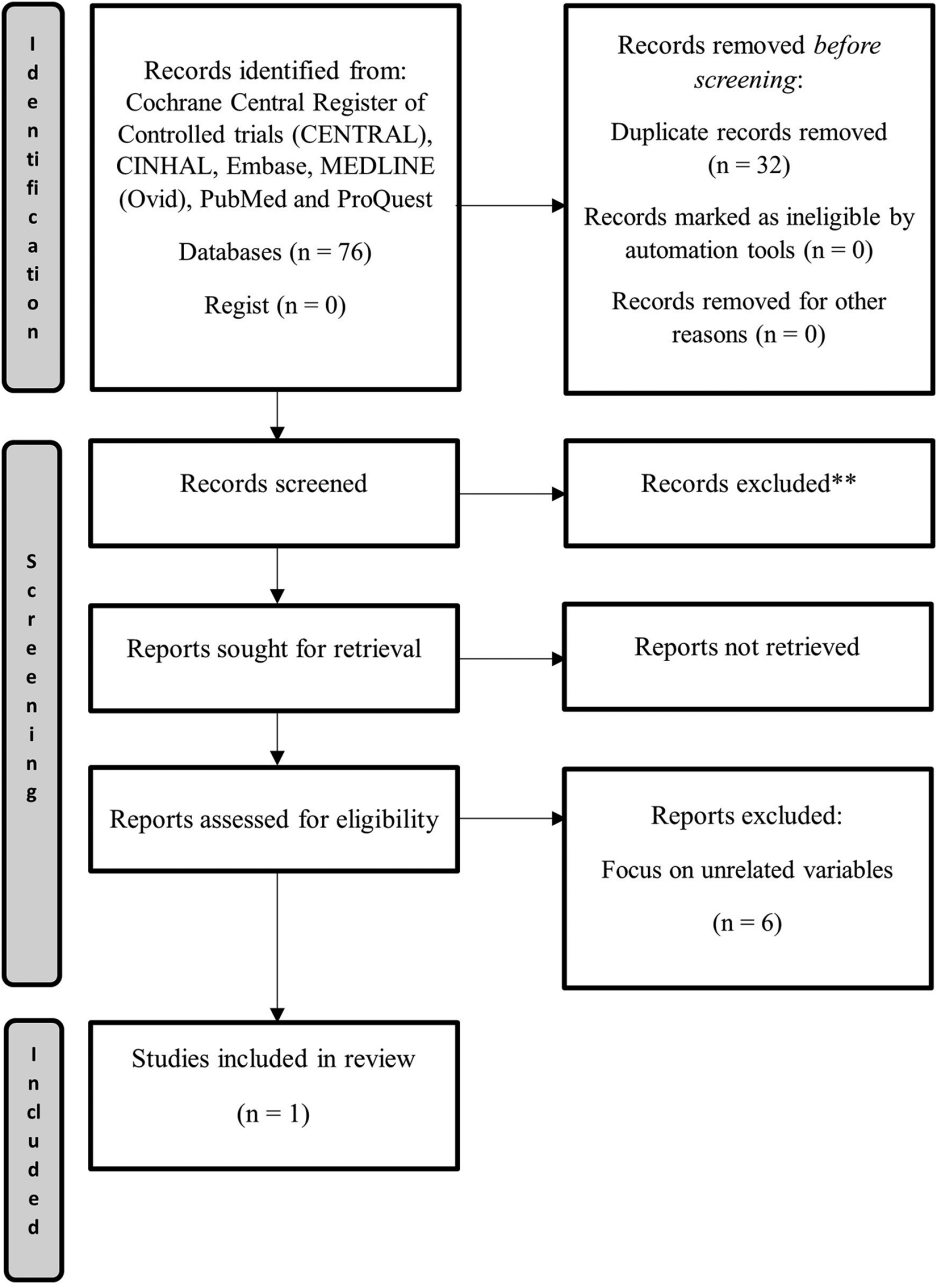
Systematic review flowchart.

Through database searches, 44 potential studies were detected without any duplicates. All of those studies were screened for relevance by title and abstract. A total of 33 references were excluded as being irrelevant to the present systematic review or incompatible with inclusion and exclusion criteria. A total of 11 articles were selected for a full text analysis, from which 10 were excluded due to focus on unrelated variables, unavailability of results or ineligible study design. Only one RCT was selected for inclusion in this review. A flow chart for this systematic review is available in Figure 1. Cohen's kappa coefficient, as calculated for the level of agreement between the two independent reviewers, was 0.65 and the percentage of agreement was 97.7%.

The only included study was a RCT written in English. It was evaluated for data extraction in accordance with the protocol for study design, participants' age and sex, interventions and control used, pain scale used and results. The RCT included 60 children from Canada that were randomized to either 1 g of amethocaine or tetracaine HCl (Ametop™) or 1 g of liposomal lidocaine (Maxilene™) prior to peripheral IV cannulation or venipuncture. Of the 60 patients included, 56 completed the study (28 in each group).

### 3.2 Risk of bias assessment

The included study was assessed for bias and was classified as *low* on all six parameters and both reviewers agreed on this assessment. A diagram depicting the final risk of bias assessment across all parameters can be seen in Table 1.

**Table 1:** Analysis of risk of bias.

Study	Sequence generation	Concealment of allocation	Blinding of results	Data of incomplete results	Selective reports	Other sources of bias
Poonai et al. (23)	Low	Low	Low	Low	Low	Low

### 3.3 Difference in mean pain score

The difference in mean pain score was assessed in the Poonai et al. (23) study, defining the outcome as change in mean pain score from baseline using the FPS-R, a self-reported pain scale that can be used reliably in children older than 4 years. Data from this study was analyzed. Overall, there was no statistically significant difference between the amethocaine and lidocaine groups on the mean difference in pain score with a mean and standard deviation (SD) of 3.7 + 3.4 and 4.3 + 3.6 (*P* = 0.28), respectively. With only one RCT study included in this review, no meta-analysis could be performed.

### 3.4 Self-reported anxiety/distress

The only included study did not report on the outcome of self-reported distress during cannulation following local topical anesthesia. Therefore, we were unable to draw any conclusions based on the available evidence.

However, the study did report on physiological characteristics associated with stress/anxiety such as heart rate. Both interventions resulted in a mean difference in heart rate (SD) of 11.2 (11.7) points for the amethocaine group and 8.5 (13.9) for the lidocaine group (*P* = 0.40), indicating no statistically significant difference.

### 3.5 Satisfaction of healthcare workers

No report was found in the included study on the outcome of healthcare workers' satisfaction during cannulation following the use of local topical anesthesia. Therefore, we were unable to draw any conclusions based on the available evidence.

However, cannulation difficulty was self-reported by the nurse performing the blood work using the Numeric Rating Scale (NRS) where 0 represents easy cannulation and 10 represents difficult cannulation. Both interventions resulted in a mean difference in cannulation difficulty of 1.8 (2.3) for amethocaine and 2.8 (2.9) for lidocaine (*P* = 0.16).

### 3.6 Children and/or parents' satisfaction

Children and/or parents' satisfaction was not measured in the Poonai et al (23). study. Therefore, we were unable to draw any conclusions based on the available evidence.

### 3.7 Occurrence of adverse events

The occurrence of adverse events was assessed in the included study (23). A transient skin reaction or an erythematous rash was noted in 10.7% of the children in the lidocaine group compared to 25% in the amethocaine group. The difference between the groups was not statistically significant (*p* = 0.3). No severe adverse event, such as methemoglobinemia, were reported in either group.

### 3.8 Success rate of the needle-related procedure

The success rate of IV cannulation was assessed in the included study (23). In the liposomal lidocaine group, success of the procedure was observed in 71.4% of children on the first cannulation attempt, 21.4% required two attempts, and 3.6% required a third attempt. In the amethocaine group, success of the procedure was observed in 82.1% of children on the first cannulation attempt, 14.3% required two attempts, and 3.6% required a third attempt. These differences were not statistically significant (*p* = 0.88). There was also no statistically significant difference between groups on mean duration of procedure. The liposomal lidocaine group had a mean procedural time of 23.9 s (SD 18.5) and the amethocaine group had a mean duration of 10 s (SD 56.4), (*p* = 0.28).

## 4 Discussion

The purpose of our systematic review was to evaluate the efficacy of two specific anesthesia cream use, amethocaine and liposomal lidocaine, in improving pain management related to needle procedures in children and adults. Despite a comprehensive literature search, we identified only one small number of studies that met our inclusion criteria. The majority of found studies consisted of small sample sizes, high biases, low methodological quality, or did not report outcomes that could be included in our systematic review. As a result, we were unable to draw any definitive conclusions about the efficacy of one anesthetic cream over the other.

The only study included in this systematic review was a RCT. This study did not report any statistically significant improvements in any of the outcomes evaluated in participants who received either amethocaine or liposomal lidocaine. Our findings cannot be compared to other similar studies as interventions are too different. In the literature, multiple types of creams have been used in other studies and included in systematic reviews, but amethocaine and liposomal lidocaine, our compounds of interest, are not often compared together on their efficacy and are most of the time compared alone to another topical anesthetic cream such as lidocaine/prilocaine cream.

The limited evidence available suggests further research is needed to confirm the superiority of one cream over the other. In particular, a well-designed RCT with a large sample size, comparing amethocaine to liposomal lidocaine as single intervention is needed to provide more conclusive evidence. The authors of the included study also agreed with this statement, noting that a major limitation of their study is the small sample size and that statistically significant differences may have arisen with more participants (23).

There are limitations to our review that may have contributed to the inconclusive findings. Firstly, the small number of studies available for inclusion in our review limits the strength of our conclusions and makes it difficult to compare the results across studies. Secondly, while the quality of the included study was high, this represents a small sample size. Moreover, excluded studies were generally of low methodological quality with many studies having a high risk of bias as well as a small sample size.
